# Luminal Chemosensory Cells in the Small Intestine

**DOI:** 10.3390/nu13113712

**Published:** 2021-10-22

**Authors:** Andreanna Burman, Izumi Kaji

**Affiliations:** 1 Cell and Developmental Biology and Epithelial Biology Center, Vanderbilt University School of Medicine, Nashville, TN 37232, USA; andreanna.j.burman@Vanderbilt.Edu; 2 Epithelial Biology Center and Section of Surgical Sciences, Vanderbilt University Medical Center, Nashville, TN 37232, USA

**Keywords:** small intestine, GPCR, enteroendocrine cell, tuft cell, enteroid

## Abstract

In addition to the small intestine’s well-known function of nutrient absorption, the small intestine also plays a major role in nutrient sensing. Similar to taste sensors seen on the tongue, GPCR-coupled nutrient sensors are expressed throughout the intestinal epithelium and respond to nutrients found in the lumen. These taste receptors respond to specific ligands, such as digested carbohydrates, fats, and proteins. The activation of nutrient sensors in the intestine allows for the induction of signaling pathways needed for the digestive system to process an influx of nutrients. Such processes include those related to glucose homeostasis and satiety. Defects in intestinal nutrient sensing have been linked to a variety of metabolic disorders, such as type 2 diabetes and obesity. Here, we review recent updates in the mechanisms related to intestinal nutrient sensors, particularly in enteroendocrine cells, and their pathological roles in disease. Additionally, we highlight the emerging nutrient sensing role of tuft cells and recent work using enteroids as a sensory organ model.

## 1. Introduction

The small intestine is a major site of nutrient absorption. Enterocytes, the largest population of small intestinal epithelium, show dynamic changes in nutrient transporter expression in response to luminal nutrient composition, suggesting that nutrient absorption mechanisms have the flexibility to adapt to nutrient availability. Chemosensory molecules in the intestinal mucosa recognize luminal nutrients and activate different signaling pathways to regulate enterocyte function, digestive functions (bile and exocrine pancreas secretion), systemic glucose homeostasis (insulin secretion), and satiety [1,2]. A variety of G protein-coupled receptors (GPCRs) are localized in different types of intestinal epithelial cells, in particular, sensory enteroendocrine and tuft cells. Enteroendocrine cells (EECs) scattered through the intestinal epithelium produce a variety of gut hormones that play important roles in the signal transduction of those physiological responses. The first section of this review summarizes numerous studies on different types of nutrient receptors and their functions, with a focus on EECs.

Another rare population of sensory cell is the tuft cell, which possesses similar sensing machinery as taste receptor cells seen in the tongue. Intestinal tuft cell had been considered as a subpopulation of EECs, however, recently identified tuft cell markers have revealed that tuft cell is a different cell lineage than other types of epithelial cells. Although several chemoreceptors are identified in tuft cells, their function as nutrient sensors has not been fully understood. Latter section of this review summarizes recent findings on intestinal tuft cells and discuss possible strategies to study sensory cells using enteroid models. The nomenclatures of receptors are following the current NCBI Gene database.

## 2. Nutrient Sensors in Enteroendocrine Cells (EECs)

### 2.1. Sugar-Sensing Receptors

In the mouth and intestine, complex carbohydrates (i.e., polysaccharides) are broken down into oligosaccharides and/or simple sugars (glucose, galactose, and fructose) that are then detected by carbohydrate-sensing molecules in the small intestinal mucosa. There are several well-characterized glucose-sensing proteins, including the sweet taste receptor (TAS1R2+TAS1R3, also known as T1R2+T1R3) and sodium-coupled glucose cotransporter-1 (SGLT1, a gene product of SLC5A1). TAS1R2 and TAS1R3 mRNA expression was first detected in the mouse small intestine by Dyer et. al, with expression of both receptor subunits being highest in the jejunum [3]. The authors also found a similar pattern of protein expression in intestinal epithelial cell membranes from the proximal, mid, and distal intestine. They also showed that the sequence of intestinal TAS1R1–3 is homologous to the mouse sequences of TAS1R1–3 found in lingual taste buds. Later, T1R1–3 expressions were confirmed throughout the human intestine [4]. TAS1R2+TAS1R3 is a mammalian heterodimeric taste receptor that is expressed in EEC cell lines, such as GLUTag and STC-1 [5,6]. Like naturally occurring sugars, non-nutritive sweeteners have high affinities for TAS1R2+TAS1R3 [6,7].

The activation of TAS1R2+TAS1R3 promotes the secretion of a variety of EEC-derived hormones, such as peptide 1 (GLP-1) and gastric inhibitory peptide (GIP). Inhibition of T1R3 in human L cells and T1R3 KO mouse intestines demonstrate a reduction in sugar-stimulated GLP-1 expression [8,9]. Similarly, T1R2 KO mice have reduced plasma GLP-1 expression following a intragastric glucose administration compared to wild-type [10]. In GLUTag cells, a mouse L cell line, the inhibition of sweet taste receptor activity by gurmarin blocked sucralose-mediated GLP-1 and GIP secretion [6]. Studies in healthy human subjects suggest similar findings, as the administration of lactisole, a sweet taste receptor antagonist, reduced intragastric and intraduodenal glucose-mediated secretion of GLP-1 and PYY [11,12]. Interestingly, the role of TAS1R2+TAS1R3 in CCK secretion is less clear. Nasogastric administration of artificial sweeteners led to an increase in plasma CCK, however, in another study, lactisole administration did not affect glucose-induced CCK secretion in human subjects [12,13]. Neither of these studies assessed TAS1R2 or 3 expression and there is a lack of literature studying the effects of TAS1R2 and 3 activities on CCK secretion by EECs.

**Figure 1:**
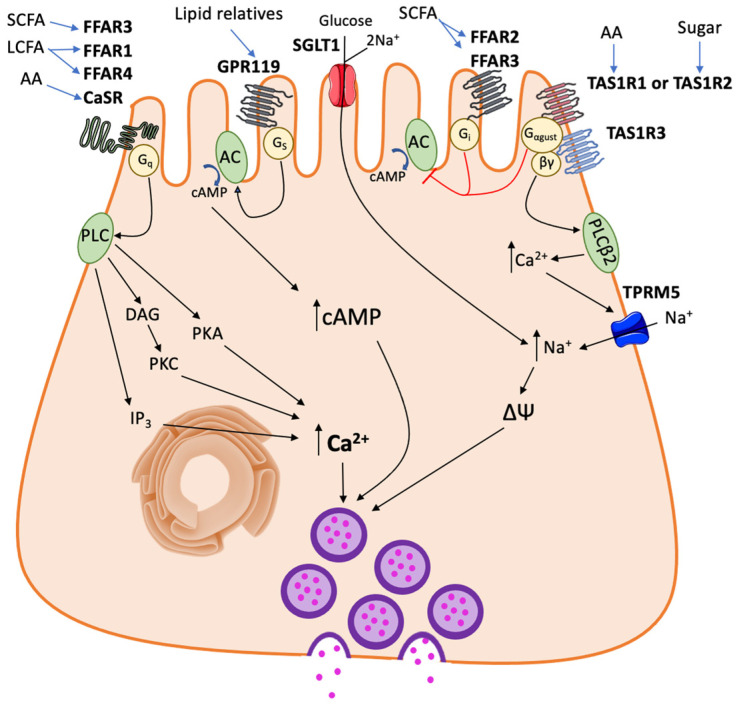
A schematic diagram of nutrient sensor signaling in an enteroendocrine cell. AA, amino acid; AC; adenylyl cyclase; cAMP, cyclic adenosine monophosphate; CaSR, calcium sensing receptor; DAG, diacylglycerol; FFAR, free fatty acid receptor; GPR119, G-protein receptor 119; IP3, inositol trisphosphate; LCFA, long chain fatty acid; PKA, protein kinase A; PKC protein kinase C; PLC, phospholipase C; PLCβ2, phospholipase C β2; SCFA, short chain fatty acid; SGLT1, sodium/glucose cotransporter 1; TAS1R, taste 1 receptor; TPRM5, transient receptor potential cation channel subfamily M member 5.

To promote the release of these peptides, TAS1R2+TAS1R3 acts with a heterotrimeric G protein, gustducin (Figure 1). The association of T1R2+3 with gustducin was first found in lingual taste cells, and their colocalization was later identified in the human duodenum via immunofluorescence [6,14]. Gustducin is made up of three components: α-gustducin (Gα_gust_, a gene product of GNAT3), Gβ3, and Gγ13. Intestinal Gα_gust_ expression was first evaluated by PCR and immunostaining of the rat duodenum [15]. Later, PCR and Western blotting revealed that Gα_gust_ is expressed throughout the small intestine (highest in the jejunum) and in STC-1 cells [3]. As a G_i/o_-coupled GPCR, the Gα_gust_ inhibits adenylyl cyclase (AC) activity, leading to a decrease in cAMP concentration [16]. Meanwhile, the βγ subunits activate phospholipase Cβ2 (PLCβ2), which catalyzes the formation of inositol triphosphate (IP3) to promote an increase in intracellular calcium concentrations [17]. This increase in calcium thereby activates TRPM5 to allow cation (i.e., Na^+^) entry into the cell [18,19]. Gustducin mediates GLP-1 release by the gut, as siRNA for Gα_gust_ prevents sugar-induced GLP-1 secretion in human L cells [8]. The release of peptide hormones, such as GLP-1, by EECs target nearby enterocytes and afferent nerves to carry out signaling activity. However, the direct connection between the TAS1R2+TAS1R3 heterodimer and incretin expression is controversial, as the intraluminal administration of non-nutritive sweeteners (TAS1R2+TAS1R3 agonists) did not promote expression of either hormone in isolated rat intestine [20]. On the other hand, mice lacking TAS1R3 have deficiencies in GLP-1 expression and insulin regulation, and sweet taste receptor antagonist administration reduces glucose-induced GLP-1 and PYY secretion [9,11]. Mice lacking TAS1R2 have deficiencies in GLP-1 and -2 expression as well as glucose absorption, due to a lack of GLP-2-induced GLUT2 translocation [10].

In addition to its traditional transporter niche, SLGT1 has an implied role in glucose sensing in the small intestine due to its expression pattern in the intestine and mediation of glucose-induced incretin section [21,22,23,24]. Dietary and artificial sugar responsive SGLT1 upregulation is dependent on TAS1R3 and Gα_gust_, as demonstrated in knockout mouse models [6]. In mice lacking SGLT1, secretion of glucose-responsive GIP and GLP-1 was diminished [25]. On the other hand, studies in diabetic rats chronically treated with selective SGLT1 inhibitors and SGLT1 knockout mice demonstrated the opposing effect of GLP-1, as inhibition of SGLT1 promoted GLP-1 secretion [26,27]. Interestingly, GLP-2 upregulates SGLT1 expression and GLP-2 promotes the relocation of glucose transporter GLUT2 (a gene product of SLC2A2) to the apical membrane in enterocytes to enhance sugar absorption [28,29]. The role of GLUT2 as a glucose sensor has been previously investigated. Studies in favor of GLUT2 as a sugar sensor emphasized the transporter’s importance in sugar-induced GLP-1 secretion using isolated rat intestine [30] and GLUT2 knockout mice [31,32]. However, a recent study using GLUT2 knockout mice demonstrated that sugar-induced GLP-1 expression did not differ between knockout and wild-type mice [25]. As mentioned by the authors, the discrepancy between the two results could be due to differences in fasting length as well as dose and route of administration of glucose.

The role of sugar sensors and their associated signaling has been explored in a variety of health conditions, including diabetes, obesity, and pathogen-induced diarrhea. As Type 2 diabetes (T2D) is a condition concerning insulin malfunction and GLP-1 secretion is involved in insulin release, the role of TAS1R2+TAS1R3 in the disorder has been explored. Although there was no difference seen in the copy number of sweet taste receptor transcripts between duodenal biopsies from T2D patients and healthy controls, the transcript number of sweet taste receptor molecules (TAS1R2, TAS1R3, Gα_gust_ and TRPM5) was inversely correlated with blood glucose levels in T2D patients [33]. Sugar sensors have also been investigated in mouse models of obesity and obese patients. Much of the research has focused on GLP-1 expression, where obese patients have reduced postprandial GLP-1 levels [34]. However, a recent study in rats have found that, despite having significantly different plasma GLP-1 concentrations, high-fat diet (HFD)-fed rats did not have a significant difference in sweet taste receptors in the intestine compared to standard diet-fed rats [35]. Interestingly, rabbits with *Escherichia coli* (*E. coli*)-induced diarrhea had a decrease in mortality and improvement of symptoms after consuming stevia leaf extract, likely mediated through TAS1R1+TAS1R3-induced upregulation of intestinal SGLT1 [36]. TAS1R2+TAS1R3 regulation in other diarrheal disorders could be of interest in future research.

### 2.2. Protein-Sensing Receptors

As proteins are broken down in the stomach by peptidases and HCl, protein sensing in the intestine involves receptors responsive to amino acids and short peptides. There are multiple amino acid sensing receptors seen in the intestine, including TAS1R1+TAS1R3 dimer [3,4], extracellular Calcium sensing receptor (CaSR) [37,38,39], GPCR class C group 6 member A (GPRC6A) [40,41], metabotropic glutamate receptors (mGluRs) [42,43], and lysophosphatidic acid receptor 5 (LPAR5, previously GPR93) [44,45]. These receptors differ in amino acid/peptide ligand preference. mGluRs specifically respond to _L_-glutamate and their signaling promotes food digestion [46,47]. LPAR5/GPR93 is a peptone receptor found in the EECs that induces CCK secretion in STC-1 cells [48,49]. GPRC6A preferentially binds to basic _L_-amino acids and is primarily expressed in intestinal epithelial cells and EECs [50,51]. GPRC6A induces GLP-1 expression in GLUTag cells when treated with _L_-ornithine [52]. A canonical calcium sensor, CaSR, is expressed throughout the intestinal epithelium (primarily in EECs) and colon, and has demonstrated peptide-sensing ability in the intestine [38]. Beyond calcium, CaSR primarily responds to basic amino acids and oligopeptides.

TAS1R1+TAS1R3, like the sweet taste receptor TAS1R2+TAS1R3, functions as a heterodimeric GPCR that binds to a broad spectrum of amino acids [7,53] and acts through coupling with α-gustducin and α-transducin [54] (Figure 1). TAS1R3 shows similar transcription patterns to that of TAS1R2, with the highest expression in the proximal small intestine, while TAS1R1 is seen in similar levels throughout the intestine [3,4]. When activated by _L_-Phe, _L_-Leu, or _L_-Glu, TAS1R1+TAS1R3 promotes CCK secretion by STC-1 cells [55]. Conversely, a recent study has demonstrated that activation of GPR35, GPR93, GPR142, and the TAS1R1+TAS1R3 do not stimulate GLP-1 release in perfused rat proximal small intestine [56]. These authors only found that CaSR promotes GLP-1 secretion. However, as indicated by these authors, the use of specific inhibitors is needed to strengthen these results. CaSR promotes oligopeptide-inducible GLP-1 secretion by primary mouse L cells in an oligopeptide transporter (PEPT)-dependent manner [57]. CaSR also functions as an aromatic amino acid sensor, where the receptor promotes CCK and GLP-1 expression in the presence of _L_-Phe in isolated I cells and in rats [58,59]. CaSR is predominantly Gα_q_ coupled, however it is known to couple with other G proteins [60,61]. Gα_q_ activates phospholipase C to promote the synthesis of diacylglycerol (DAG) and IP3. Together, DAG and IP3 induce an increase in intracellular calcium for signaling events.

In addition to the different receptors, the signaling outcome of amino acid signaling is dependent on the specific amino acid ligand and its location (luminal vs. vascular side). For example, L-Val induces the greatest GLP-1 release on the luminal side of perfused rat intestine, while L-Arg and aromatic amino acids do so on the vascular side [56].

Contrary to carbohydrates, intestinal protein sensing response remains unchanged in obesity. This finding is observed as obese individuals have similar CCK and GLP-1 levels in response to intraduodenal protein infusion to what is seen in lean individuals [62]. Beyond this, protein intake reduces postprandial glucose response in T2D individuals and improvement in body composition in obese subjects [63,64]. Similar to sweet taste receptors [36], CaSR also has implicated therapeutic potential in alleviating diarrhea in isolated rat intestines, as the receptor is a modulator of intestinal fluid secretion and absorption [65,66]. However, these findings have only been reported in calcium-activated CaSR, and protein activation of CaSR has yet to be explored in these aspects.

### 2.3. Fatty Acid-Sensing Receptors

Dietary fats, typically triglycerides, are primarily broken down by lipases in the intestinal lumen into free medium- or long-chain fatty acids (LCFAs) prior to nutrient detection. SCFAs are ligands for free fatty acid receptor (FFAR) 2 (previously GPR43) and FFAR3 (GPR41), while LCFAs are detected by FFAR1 (GPR40), GPR119, and FFAR4 (GPR120) (Figure 1). Similar to protein sensors, the different FA receptors have specific ligand preferences [67]. For example, FFAR2 has equal potency with acetate and propionate, while FFAR3 has increased potency with propionate compared to acetate [68]. FFAR1 preferentially binds to pentadecanoic and palmitic acids, while FFAR4 demonstrates greatest potency with saturated FAs between 14–18 carbons in length and unsaturated free FAs between 16–22 carbons in length [69,70]. All four FFARs are expressed in L cells [71,72]. FFAR4 is primarily expressed in EECs of ileum [69].

Despite their similar length of FA ligands, FFAR2 and FFAR3 are coupled with different G proteins, with FFAR2 coupled to G_i/o_ proteins and FFAR3 having dual coupling with G_i/o_ and G_q_ proteins [73]. Both receptors primarily promote their signaling activity though IP3-induced intracellular calcium release. FFAR2 mediates metabolic homeostasis through promoting leptin secretion, a lipogenesis inhibitor [74]. In contrast, FFAR3 inhibits lipolysis and its signaling lessens plasma FA levels in vivo [75]. FFAR1 and FFAR4 are coupled to G_q_ and are involved in insulin synthesis via incretin secretion signaling as demonstrated in STC-1 cells and mice [69,70,76,77].

GPR119 is another fatty acid sensor, which primarily interacts with phospholipid and fatty acid amide ligands [78,79]. GPR119 is selectively coupled with Gs, as the receptor acts through increasing cAMP concentrations by promoting adenylyl cyclase (AC) activity [80].

The cluster of differentiation 36 (CD36) is a fatty acid transporter with FA-sensing abilities. As a scavenger receptor, CD36 interacts with a variety of ligands, including LCFAs [81]. CD36 is expressed on the brush border of enterocytes and EECs primarily in the proximal intestine [82,83]. CD36 activity in enterocytes has been linked to prolonging satiety though mediating the conversion of FA into oleoylethanolamide, as demonstrated in knockout mice [84]. In EECs, CD36 activation promotes the CCK secretion via cAMP activation and secretin secretion via cAMP-PKA signaling, as demonstrated in knockout mice [85]. CD36 is also involved in GLP-1 and GIP secretion, as obese African American women carrying a CD36-diminishing mutation release fewer incretins after ingesting a high-fat meal compared to controls [86]. Interestingly, the stimulation of anorectic signals by dietary fat is dependent of chylomicron formation from FAs in rats, indicating that fat sensing also occurs on the basolateral side of the intestine following FA absorption [87]. To this end, CD36 could support the hypothesis of basolateral fat sensing through its FA transport activity and promotion of chylomicron synthesis [88].

Similar to amino acid- and sugar-sensing, the role of fat-sensing in obesity and diabetes has been studied. Interestingly, duodenal CD36 and FFAR4 transcript levels are directly correlated with BMI in lean and obese subjects [89]. However, small intestinal *Cd36* expression is significantly downregulated in high sucrose diet (HSD)-fed mice, but high-fat + high-sucrose (HFHSD)-fed mice have elevated CD36 transcript levels [90,91]. These studies indicate that body weight alone is not completely indicative of CD36 expression, and diet composition must be considered. The other FFARs, particularly FFAR1, have been studied as therapeutic targets for metabolic disorders. Using a combination treatment of FFAR1 agonists and DPP-IV (incretin degradation enzyme) inhibitors, insulin secretion and glucose metabolism were improved in *ob/ob* mice [92].

A variety of sugar-, amino acid-, and fatty acid-sensing molecules are expressed in specific cells of the intestinal epithelium that initiate physiological responses to luminal nutrients.

## 3. Sensory Tuft Cell in the Small Intestine

### 3.1. Similarity of Intestinal Tuft Cell and Lingual Taste Receptor Cell

**Figure 2:**
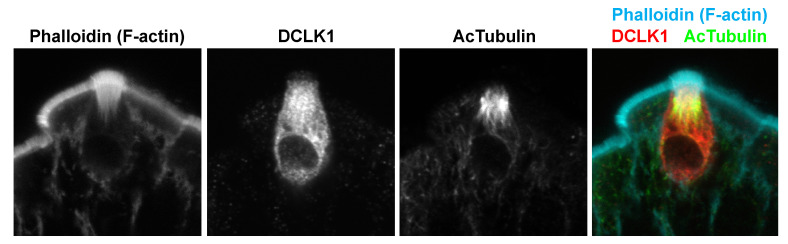
Tuft cell morphology in mouse small intestine. Widely used tuft cell markers, F-actin (phalloidin), DCLK1, and acetylated tubulin demonstrate the microvillus structure, whole-cell shape, and dense microtubules, respectively.

Over the past decade, the intestinal tuft cell has been acknowledged as a different cell lineage from other types of epithelial cell, including EEC. Through electron microscopy, the distinct ultrastructure of long, stiff microvilli and the well-developed tubulovesicular system were identified in the 1950s in tuft cells, also known as brush, caveolated, multivesicular, or fibrillovesicular cells [93,94]. However, intestinal tuft cells had not been characterized well until doublecortin-like kinase 1 (DCLK1) was identified as a tuft cell-specific marker in mice [95]. In later histochemical studies, the jelly fish-like apical tuft structure has been described with the actin-associating phalloidin, gamma-actin, and advillin, but not villin unlike other types of intestinal epithelial cells [95,96,97] (Figure 2). In addition to morphological similarity, intestinal tuft cells possess chemosensory and signal transduction machineries that are seen in lingual taste receptor cells.

Sweet, umami, or bitter compounds in foods are detected by different taste receptor cells that express distinct GPCRs, namely TAS1R2+TAS1R3, TAS1R1+TAS1R3, or TAS2R(s), respectively. A master transcription factor POU2F3 (POU Class 2 Homeobox 3) generates these taste receptor cells in the taste buds [98]. Pou2f3-deficient mice specifically lack tuft cells in the intestine, whereas other cell lineages are equivalent to that in wild-type mice [99]. Taste transduction molecules are commonly expressed in tuft and taste receptor cells alike in mice, including GNAT3, TRPM5, PLCβ2, and TAS1R members [4,15]. Single cell RNA-sequencing studies have revealed that mouse intestinal tuft cells express a variety of GPCRs, including *Sucnr1 (Gpr91)*, *Ffar3*, *Gprc5c*, and some orphan GPCRs [100]. These transcription signatures suggest that tuft cells have the sensing ability for succinate (SUCNR1), short-chain fatty acids (FFAR3), acid/base balance (GPRC5C), and other unknown ligands in the lumen. Unlike the GPCRs for endogenous hormones that show high affinity (<100 nM), the GPCRs expressed on taste buds and intestinal tuft cells require 0.1–100 mM range of ligands and respond to broad spectrum of chemicals with similar structure [101]. This range is suitable to nutrient concentrations in the intestinal lumen. Individual tuft cell may express a limited number of GPCRs and respond to distinct ligands similar to that of the lingual taste receptor cells [98]. In contrast to the direct communication of taste receptor cells with gustatory nerves, intestinal tuft cells likely release IL-25, acetylcholine (ACh), and/or eicosanoids (prostaglandins and leukotrienes) in paracrine fashion to neighboring cells or nerves.

### 3.2. Intestinal Tuft Cell Markers

**Figure 3:**
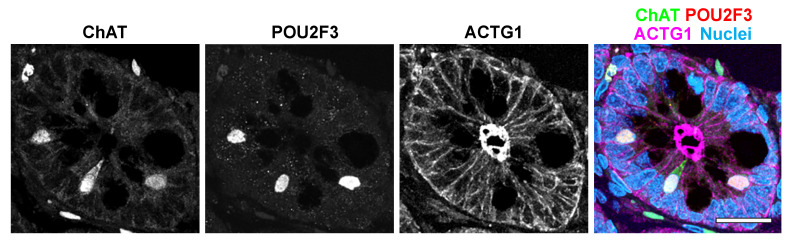
Immunostaining for human intestinal tuft cells. Both ChAT and POU2F3 are detected in tuft cells in jejunum paraffin sections. Scale bar: 20 µm.

Most POU2F3-expressing (POU2F3^+^) tuft cells express signal transmitter enzymes that indicate their signal transduction function. Immunostaining for these enzymes is used as intestinal tuft cell markers in mouse and human tissues, including choline acetyl transferase (ChAT) (Figure 3), prostaglandin synthase 1 (PTGS1, also known as COX1), PTGS2 (COX2), hematopoietic prostaglandin D synthase (HPGDS), and arachidonate 5-lypoxygenase (ALOX5) [95,102]. Tuft cell-specific tyrosine phosphorylation was identified in the epidermal growth factor receptor (EGFR)(Y1068) [103] and an actin-binding protein, CCDC88A (GIRDIN)(Y1798) [104], suggesting a distinct kinase pathway in the tuft cell. Although the majority of intestinal tuft cells possess ChAT protein, other cholinergic neuron machineries, such as vesicle ACh transporter or high-affinity choline transporter (SLC5A7), have not been identified in the tuft cell [105]. Whether ACh is actually secreted into extracellular space or is acting via intracellular signaling remains unknown. DCLK1 is also widely utilized as a tuft cell marker and Cre/loxP target in genetically engineered mice, however, the DCLK1 expression in human tuft cells has not been confirmed. Esmaeilniakooshkghazi et al. recently reported that advillin protein selectively accumulates in intestinal tuft cells of mice [96]. Since the predominate expression of advillin was identified in peripheral sensory neurons, *Advillin-Cre* mice have been utilized to target the sensory neurons [106]. To distinguish intestinal tuft cell-specific function, this mouse strain can be used in enteroid studies.

### 3.3. Tuft Cell Function in Mucosal Homeostasis

DCLK1^+^ tuft cell depletion reduces epithelial cell proliferation and survival rate of mice after mucosal injury [107,108]. These studies suggest that tuft cells may support stem cell activity and promote mucosal restitution. Indeed, our quantitative immunofluorescence studies demonstrated that tuft cell frequency was significantly decreased in celiac disease patients who showed malabsorption [109]. Several clinical trials have been reported, demonstrating that experimental helminth infections suppress inflammatory cytokines and allergic reactions in patients with autoimmune disorders, including celiac disease and inflammatory bowel disease (IBD) [110,111,112]. In mouse experimental models, helminth inoculation induces tuft cell hyperplasia and activates type 2 immune response through IL-25 secretion from tuft cells [99,113]. Tuft cell contribution to the type 2 immune response is reviewed elsewhere [114,115]. Although there is no report about the change in human tuft cell population under the parasite infections, tuft cell-mediated mucosal restitution might also be important for epithelial homeostasis in humans. Furthermore, a significant decrease in tuft cell number is correlated with the inflammation severity in duodenal ulceration [109] and ileal inflammation in Crohn’s disease [116]. Loss of tuft cell population can be an indicator of impaired mucosal integrity and activating tuft cells might be a therapeutic pathway for enteropathy. As expected by *Sucnr1* transcriptional signature in intestinal tuft cells, 100–150 mM succinate in drinking water, but not short-chain fatty acids that activate FFAR3, leads tuft cell hyperplasia and type 2 immune responses via IL-25 release [117,118]. Several studies using knockout mouse strains confirmed that the succinate-induced tuft cell activation is mediated by SUCNR1, GNAT3, PLCß2, TRPM5, and TAS1R3, suggesting the same cascade as that in lingual taste receptor cells [117,118,119,120]. SUCNR1-deficient mice lack the response to protozoa-derived succinate but have intact immune response to helminth infection [119]. The sensory mechanism for helminth by the tuft cell is still unknown. Unlike mouse tissues, IL-25 production or any chemosensing receptor expression in human tuft cell has not yet been confirmed. Recently, predominant expression of Fc fragments was identified in a subset of human intestinal tuft cells, suggesting the involvement in immune response [121]. Identification of tuft cell activation pathways in human intestine might support the development of treatments for autoimmune disorders.

### 3.4. Enteroid/Intestinal Organoid as a Sensory Organ Model

Sato and Clevers established 3-dimentional enteroid culture technique in 2009, demonstrating that Lgr5^+^ stem cells can differentiate into all types of intestinal epithelial cells [122]. The term enteroid indicates primary cultures of isolated stem cell-derived epithelium, while inducible pluripotent stem (iPS) cell-derived “mini gut” cultures are termed intestinal organoids (reviewed in [123]). Enteroids recapitulate the functional characteristics of original tissues, including intestinal segmental differences and disease phenotypes, suggesting that this is a closer model to epithelial physiology than immortalized cell lines [124].

Studying luminal the chemosensing mechanism in 3-dimensional enteroid cultures has had difficulties, since the luminal volume of enteroids cannot be estimated to administer precise concentrations of compounds. The Donowitz and Zachos groups have established 2-dimensional cultures of human and mouse enteroids that allow for the measurement of epithelial ion transport and cytokine release into the luminal and basolateral spaces [125,126]. The combination of activators and/or inhibitors of Wnt and Notch signaling pathways can manipulate the cell lineage populations in enteroid systems [127]. Treatment with Notch inhibitors induces tuft cell hyperplasia in vivo and in enteroids [113,128], suggesting that tuft cell development likely requires Wnt signaling activation. We recently reported that tuft cell differentiation was specifically inhibited by myosin Vb (MYO5B) loss, a model of a congenital diarrheal disorder, microvillus inclusion disease [97]. MYO5B-deficient epithelial cells showed reduced Wnt ligands, while Notch ligands remained unchanged, thereby inducing an imbalance of Notch/Wnt signaling. A Wnt signaling inhibitor, Wnt-C59, significantly reduced tuft cell differentiation in wild-type mouse enteroids, but had no effect on MYO5B-deficient enteroids [97]. Similarly, the combination of high Wnt and a Notch inhibitor, DBZ, enhanced taste receptor cell development in gustatory organoids [129,130]. These observations suggest that a low Notch and high Wnt environment initiate chemosensory cell differentiation in the enteroid/organoid cultures. IL-4 and IL-13 supplementation increase tuft cell differentiation in mouse enteroids, at least partly mediated by Notch signaling modulation [96,99,113]. Tuft cell-enriched enteroids as monolayer on transwell inserts serves as a useful tool to identify chemoreceptors of tuft cells and to determine nutrient handling of neighboring enterocytes in response to tuft cell activation. Furthermore, immune cells or neurons can be co-cultured in the transwell system to investigate cell-cell interaction downstream of chemosensing.

The enteroid technique is a promising tool for studying tuft cell functions in healthy and disease conditions. Of note, abnormal glucose absorption and gluconeogenesis in obese patients are recapitulated in enteroids generated from these patients [131]. Enteroids could also potentially be used to model congenital diarrhea disorders. Congenital diarrheal patients with diacylglycerol acyltransferase (DGAT)1 mutation are known to be rescued by a very low-fat diet [132]. However, long-term avoidance of dietary fat induces essential fatty acid depletion that affects the development of humoral and nervous systems. Since DGAT1 knockout mice do not recapitulate the human patient phenotype, the mechanistic study for lipid-induced diarrhea in DGAT1 mutant patients is hampered. Future studies with patient enteroids may provide important clues of lipid sensing mechanism and therapeutic strategies for those patients as well as for other unclassified congenital diarrheal diseases.

## 4. Conclusions

Recent studies have revealed the correlation between intestinal nutrient sensors and metabolic, immune, and diarrheal disorders. Nutrient sensing pathways and tuft cell activation can be therapeutic targets to normalize nutrient absorption and mucosal restitution.

## Data Availability

Not applicable.
